# Genetic analysis of reaction time variability: room for improvement?

**DOI:** 10.1017/S0033291712002061

**Published:** 2012-09-14

**Authors:** J. Kuntsi, A. C. Frazier-Wood, T. Banaschewski, M. Gill, A. Miranda, R. D. Oades, H. Roeyers, A. Rothenberger, H.-C. Steinhausen, J. J. van der Meere, S. V. Faraone, P. Asherson, F. Rijsdijk

**Affiliations:** 1 King's College London, MRC Social, Genetic and Developmental Psychiatry Centre, Institute of Psychiatry, UK; 2 Department of Epidemiology and Section on Statistical Genetics, University of Alabama at Birmingham, AL, USA; 3 Department of Child and Adolescent Psychiatry and Psychotherapy, Central Institute of Mental Health, University of Heidelberg, Mannheim, Germany; 4 Department of Psychiatry, Trinity Centre for Health Sciences, St James's Hospital, Dublin, Ireland; 5 Department of Developmental and Educational Psychology, University of Valencia, Spain; 6 Clinic for Child and Adolescent Psychiatry and Psychotherapy, University of Duisburg-Essen, Essen, Germany; 7 Department of Experimental Clinical and Health Psychology, Ghent University, Belgium; 8 Child and Adolescent Psychiatry, University of Göttingen, Germany; 9 Department of Child and Adolescent Psychiatry, University of Zurich, Switzerland; 10 Clinical Psychology and Epidemiology, Institute of Psychology, University of Basel, Switzerland; 11 Research Unit for Child and Adolescent Psychiatry, Psychiatric Hospital Aalborg, University Hospital Aarhus, Denmark; 12 Department of Developmental and Clinical Neuropsychology, University of Groningen, The Netherlands; 13 Department of Neuroscience, SUNY Upstate Medical University, Syracuse, NY, USA; 14 Department of Psychiatry, SUNY Upstate Medical University, Syracuse, NY, USA

**Keywords:** ADHD, event rate, genetic effects, reaction time variability, rewards

## Abstract

**Background:**

Increased reaction time variability (RTV) on cognitive tasks requiring a speeded response is characteristic of several psychiatric disorders. In attention deficit hyperactivity disorder (ADHD), the association with RTV is strong phenotypically and genetically, yet high RTV is not a stable impairment but shows ADHD-sensitive improvement under certain conditions, such as those with rewards. The state regulation theory proposed that the RTV difference score, which captures change from baseline to a rewarded or fast condition, specifically measures ‘state regulation’. By contrast, the interpretation of RTV baseline (slow, unrewarded) scores is debated. We aimed to investigate directly the degree of phenotypic and etiological overlap between RTV baseline and RTV difference scores.

**Method:**

We conducted genetic model fitting analyses on go/no-go and fast task RTV data, across task conditions manipulating rewards and event rate, from a population-based twin sample (*n*=1314) and an ADHD and control sibling-pair sample (*n*=1265).

**Results:**

Phenotypic and genetic/familial correlations were consistently high (0.72–0.98) between RTV baseline and difference scores, across tasks, manipulations and samples. By contrast, correlations were low between RTV in the manipulated condition and difference scores. A comparison across two different go/no-go task RTV difference scores (slow-fast/slow-incentive) showed high phenotypic and genetic/familial overlap (*r* = 0.75–0.83).

**Conclusions:**

Our finding that RTV difference scores measure largely the same etiological process as RTV under baseline condition supports theories emphasizing the malleability of the observed high RTV. Given the statistical shortcomings of difference scores, we recommend the use of RTV baseline scores for most analyses, including genetic analyses.

## Introduction

Increased reaction time variability (RTV) on cognitive tasks requiring a speeded response is characteristic of several psychiatric disorders, including attention deficit hyperactivity disorder (ADHD; Kuntsi & Klein, 2012), schizophrenia (Kaiser *et al.* 2008) and bipolar disorder (Brotman *et al.* 2009).

High RTV in ADHD has in particular attracted a large number of studies, which serve as a useful example of how to uncover the nature and etiology of this phenomenon. The starting point has been the strong association of ADHD with high RTV, which has been replicated across many tasks, samples and definitions of ADHD (as a diagnosis or a continuum of symptoms) (Kuntsi & Klein, 2012). In a large-scale ADHD and control sibling-pair study we recently found evidence for a familial RT cognitive impairment factor, capturing RTV and overall slower RTs, that accounted for around 85% of the familial influences on ADHD, and separated from a smaller familial factor that captured commission and omission errors (Kuntsi *et al.* 2010).

Several possible explanations for high RTV in ADHD are under investigation (Castellanos *et al.* 2009; Yordanova *et al.* 2011; Kuntsi & Klein, 2012), and these can be roughly divided into those that consider RTV to reflect a stable impairment and those that emphasize the malleability of the observed high RTV. The proposal that ADHD reflects arousal regulation difficulties that lead to a vigilance decrement, based on the state regulation/cognitive-energetic model (van der Meere, 2002; Sergeant, 2005), predicts that RTV should not be stable in ADHD but should improve in conditions that successfully optimize the state of the child. Supporting the predictions, in both an ADHD and control sibling-pair sample and a population-based twin sample, we have observed a greater difference in RTV among ADHD than the control group, following the introduction of rewards or rewards with a faster event rate (Andreou *et al.* 2007; Kuntsi *et al.* 2009; Uebel *et al.* 2010), consistent with earlier reports (Slusarek *et al.* 2001). These findings are inconsistent with the possibility that high RTV in ADHD stems from a stable impairment, reflecting non-specific brain trauma. Furthermore, the etiological influences that ADHD shares with those on RTV largely separate from the etiological influences that ADHD shares with IQ (Rommelse *et al.* 2008; Wood *et al.* 2010, 2011). Although these theoretical approaches have been developed in relation to ADHD, they could be applied to study increased RTV in other disorders.

The original state regulation model (van der Meere, 2002) provides further testable hypotheses. It proposes that the high RTV in ADHD under baseline (slow, unrewarded) conditions reflects poor state regulation, and views the change in RTV from baseline to a rewarded or faster condition as a specific measure of state regulation. That is, whereas high RTV under baseline conditions can be explained by several alternative models, including those linking RTV to a stable brain impairment, the RTV difference score is proposed specifically to measure state regulation. In the state regulation model, rewards and event rate are further proposed to partially influence different pathways, but relevant evidence is limited.

We aimed to investigate directly the degree of phenotypic and etiological overlap between RTV baseline and RTV difference scores (difference from baseline to a rewarded and/or faster condition; see Table 1). Strong phenotypic and etiological overlap between RTV baseline and difference scores would indicate that baseline RTV captures the same underlying process as the RTV difference scores. Conversely, we predicted a less strong phenotypic and etiological overlap between RTV in the manipulated condition (rewards and/or faster event rate) and RTV difference scores. In other words, we predicted that individuals with high RTV in a baseline condition would show greater potential for improving their RTV, whereas RTV in a manipulated condition would not be strongly associated with their potential for improving RTV because it measures their best possible RTV performance. Our second main aim was to investigate the phenotypic and etiological overlap between RTV difference scores obtained using reward *versus* event rate manipulations. Again, strong phenotypic and etiological overlap across the two RTV difference scores would indicate a shared underlying process whereas low correlations would suggest separable processes. By carrying out identical quantitative genetic analyses on a population-based twin sample and an ADHD and control sibling-pair sample, we aimed to examine the generalizability of findings from a population-based sample to a clinically ascertained sample.

**Table 1.** Definition of RTV variables

RTV variable	Abbreviation	Definition
Go/no-go task		
Slow condition RTV	RTV_slow_	RTV in a condition with a slow event rate (an inter-stimulus interval of 8 s)
Fast condition RTV	RTV_fast_	RTV in a condition with a fast event rate (1 s)
Incentive condition RTV	RTV_incentive_	RTV in a condition where: one point is lost for each omission error (failure to respond to X) or failure to respond within 2 s; five points are lost for each commission error (incorrect response to O); one point is earned for each correct response
Slow-fast RTV difference score	RTV-dif_slow-fast_	Change in RTV from slow to fast condition
Slow-incentive RTV difference score	RTV-dif_slow-incentive_	Change in RTV from slow to incentive condition
Fast task		
Baseline condition RTV	RTV_baseline_	RTV in a condition with a slow event rate (8 s)
Fast-incentive condition RTV	RTV_FI_	RTV in a condition with a fast event rate (1 s) and incentives. Smiley faces are won for consistently faster responding (one for each time that a participant responds faster than their own MRT during the baseline condition consecutively for three trials)
Baseline to fast-incentive RTV difference score	RTV-dif_baseline-FI_	Change in RTV from baseline to fast-incentive condition

RTV, Reaction time variability; dif, difference score; FI, fast incentive; MRT mean reaction time.

## Method

### Sample

#### Twin sample

Participants were members of the Study of Activity and Impulsivity Levels in children (SAIL), a general population sample of twins aged 7–10 years. They were recruited from the Twins' Early Development Study (TEDS; Trouton *et al.* 2002), a birth cohort study that invited parents of all twins born in England and Wales during 1994–1996 to enroll. The TEDS families are representative of the UK population with respect to parental occupation, education and ethnicity (Oliver & Plomin, 2007).

TEDS families were invited to take part if they fulfilled the following SAIL project inclusion criteria: twins' birthdates between 1 September 1995 and 31 December 1996; lived within a feasible traveling distance from the research center; White European ethnic origin (to reduce population heterogeneity for molecular genetic studies); recent participation in TEDS, as indicated by return of questionnaires at either a 4- or 7-year data collection point; no extreme pregnancy, perinatal difficulties, specific medical syndromes, chromosomal anomalies or epilepsy; not participating in other current TEDS substudies; and not on stimulant or other neuropsychiatric medications.

Of the 1230 suitable families contacted, 672 families (55%) agreed to participate. Thirty individual children were subsequently excluded due to: IQ < 70, epilepsy, obsessive–compulsive disorder, autism or other neurodevelopmental disorder, illness during testing or placement on stimulant medication for ADHD. The final sample consisted of 1314 individuals: 255 monozygotic (MZ) twin pairs, 184 same-sex dizygotic (DZ) and 207 opposite-sex DZ twin pairs, and also 22 singletons coming from pairs with one of the twins excluded. Data for the 22 singleton twins were also used in the structural equation modeling (Neale *et al.* 2006). Participants were invited to our research center for a cognitive assessment, where ratings on the Conners' scale were collected from parents (Kuntsi *et al.* 2006). Teachers' ratings on the Conners' scale were obtained through the post. The mean age of the sample was 8.83 years (s.d. = 0.67) and half of the sample were girls (51%). The mean IQ was 109.34 (s.d. = 14.72). Parents of all participants gave informed consent following procedures approved by the Institute of Psychiatry Ethical Committee.

#### ADHD and control sibling-pair sample

##### ADHD probands and siblings

Participants were recruited from specialist clinics in Belgium, Germany, Ireland, Israel, Spain, Switzerland and the UK, through the International Multicenter ADHD Genetics (IMAGE) project (Chen *et al.* 2008). All participants were of European Caucasian descent and aged 6–18 years. All probands had a clinical diagnosis of combined subtype ADHD (ADHD-CT) and had a full sibling (unselected for clinical phenotype) and biological parents available for ascertainment of clinical information and DNA. Exclusion criteria for both probands and siblings included IQ < 70, autism, epilepsy, general learning difficulties, brain disorders and any genetic or medical disorder associated with externalizing behaviors that might mimic ADHD. Sibling selection was based, first, on gender and, second, on nearest age to the index proband.

##### Control sample

The control group was recruited from primary (ages 6–11 years) and secondary (ages 12–18 years) schools in the UK, Germany and Spain, aiming for an age and sex match with the clinical sample. The same exclusion criteria were applied as for the clinical sample. One child subsequently withdrew after testing and three were excluded for having an IQ < 70. A further 10 controls were excluded for having both parent and teacher Conners' DSM-IV ADHD subscale *T* scores >63, to exclude potential, undiagnosed ADHD cases.

##### Final sample

The ADHD proband and sibling sample consisted of 920 individuals (464 ADHD probands and 456 siblings of ADHD probands) and the control sample of 345 individuals. The final total sample therefore consisted of 1265 individuals, which comprised 580 complete sibling pairs and 105 singletons. The mean age was 11.45 years (s.d. = 2.73) for probands with ADHD, 11.38 years (s.d. = 2.96) for siblings of probands and 12.07 years (s.d. = 2.47) for controls. The percentage of males was 89.01, 49.78 and 70.43% respectively. The mean IQ was 102.02 (s.d. = 15.44) for probands with ADHD, 103.43 (s.d. = 13.59) for siblings of probands and 108.91 (s.d. = 13.71) for controls. Of the 1265 individuals, 524 with ADHD-CT were classified as affected, 16 who met criteria for the hyperactive-impulsive or inattentive subtypes were classified as a ‘subthreshold group’, and a further 664 individuals were unaffected siblings and controls. ADHD status was therefore included in the analyses in an ordinalized manner. A further 61 participants had cognitive data, but no clinical data, and their affection status was coded as missing. Some cognitive data are missing because two of the teams did not administer the go/no-go task, two did not administer the fast task, and there were occasional technical problems with equipment. Go/no-go data were available from 922 participants and fast task data from 687 participants. Of the 524 individuals with ADHD-CT, 151 had conduct disorder, 355 had oppositional defiant disorder and 63 had possible mood disorder (excluding bipolar disorder), derived as part of the PACS interview (see Measures). Ethical approval was obtained from local ethical review boards.

### Procedure

The assessments of the proband and sibling/twins in a pair were carried out in separate rooms. Short breaks were given as required and the total length of the test session was 2.5–3 h. For participants on medication for ADHD, a minimum of a 48-h medication-free period was required for cognitive testing.

### Measures

#### ADHD diagnosis

The Parental Account of Child Symptoms (PACS) interview (Taylor *et al.* 1986*a*,*b*) was conducted with the parents of the ADHD sample to derive the 18 DSM-IV symptoms for ADHD index cases plus siblings who were thought, on the basis of parents' descriptions of behavior or Conners' scores ⩾65, to have ADHD. Situational pervasiveness was defined as some symptoms occurring within two or more different situations from the PACS, along with the presence of one or more symptoms scoring ⩾2 from the DSM-IV ADHD subscale of the teacher-rated Conners' (Conners, 2003). Impairment criteria were based on severity of symptoms identified in the PACS. Across the IMAGE sites a mean κ coefficient of 0.88 and an average agreement of 96.6% were obtained for ADHD diagnostic categories (Asherson *et al.* 2008).

#### Rating scales

ADHD symptoms were measured using the Long Version of Conners' Parent Rating Scale (CPRS-R:L; Conners *et al.* 1998*a*) and the Long Version of Conners' Teacher Rating Scale (CTRS-R:L; Conners *et al.* 1998*b*). On both the parent and teacher Conners' scales, summing the scores on the nine-item hyperactive-impulsive and nine-item inattentive DSM-IV symptoms subscales forms a total DSM-IV ADHD symptoms subscale. We created an ADHD composite score by taking a mean of the scores on the parent and teacher DSM-IV ADHD symptoms subscales. In a few cases, missing data in Conners' scales were prorated (i.e. a summary score based on the mean of individual questions on the rest of the subscale was used) if there was more than 75% completion for each subscale.

#### Cognitive tasks

##### The go/no-go task (Borger & van der Meere, 2000; Kuntsi *et al.* 2005)

On each trial, one of two possible stimuli appeared for 300 ms in the middle of the computer screen. The participant was instructed to respond only to the ‘go’ stimuli and to react as quickly as possible, but to maintain a high level of accuracy. The proportion of ‘go’ stimuli to ‘no-go’ stimuli was 4:1. The participants performed the task under three conditions (slow, fast and incentive; Kuntsi *et al.* 2009), matched for length of time on task. The slow condition had an inter-stimulus interval (ISI) of 8 s and consisted of 72 trials. The ISI was 1 s in the fast condition, which consisted of 462 trials. The order of presentation of the slow and fast conditions varied randomly across participants.

The incentive condition was administered last, to ensure that the possibility of earning rewards would not adversely affect performance on the other conditions where rewards could not be earned. Each correct response to the letter X and each correct non-response to the letter O earned the child one point. The child lost one point for each omission error (failure to respond to X) and for each failure to respond within 2 s. Each commission error (incorrect response to O) led to the loss of five points. The points were shown in a box, immediately right of the screen center, and were updated continuously throughout. The child started with 40 points, to avoid the possibility of a negative tally. The child was asked to try to win as many points as possible, and was told that the points would be exchanged for a real prize when the game ended. This condition consisted of 72 trials and had an ISI of 8 s.

##### The fast task (Kuntsi *et al.* 2006; Andreou *et al.* 2007)

The baseline condition, with a fore period of 8 s and consisting of 72 trials, followed a standard warned four-choice RT task. A warning signal (four empty circles, arranged side by side) first appeared on the screen. At the end of the fore period (presentation interval for the warning signal), the circle designated as the target signal for that trial was filled (colored) in. The participant was asked to make a compatible choice by pressing the response key that directly corresponded in position to the location of the target stimulus. Following a response, the stimuli disappeared from the screen and a fixed inter-trial interval of 2.5 s followed. Speed and accuracy were emphasized equally. If the child did not respond within 10 s, the trial terminated.

A comparison condition with a fast event rate (1 s) and incentives, consisting of 80 trials, followed the baseline condition. The participants were told to respond really quickly one after another to win smiley faces and earn real prizes in the end. The participants won a smiley face for responding faster than their own mean reaction time (MRT) during the baseline condition consecutively for three trials. The baseline MRT was calculated here based on the middle 94% of responses, therefore excluding extremely fast and extremely slow responses. The smiley faces appeared below the circles in the middle of the screen and were updated continuously. For analyses that compare performance across the baseline and fast-incentive conditions, the full fast-incentive condition data are compared to baseline condition data matched for length of time on task (further details in Andreou *et al.* 2007). The variables obtained from the task are MRT and s.d. of RTs.

### Analyses

Data preparation was conducted in Stata version 9.2 (Stata Corporation, USA). All models were fitted to age- and sex-regressed residual scores. Genetic and familial structural equation models were conducted in Mx (Neale *et al.* 2006). Participants with incomplete data were included, as Mx handles missing data by using raw maximum likelihood estimation to calculate a likelihood statistic for each observation based on the observed variance/covariance matrix.

#### Modeling twin data

All residual scores were transformed to normality using the optimized minimal skew command in Stata version 9.2. Constrained correlation models were run that reflected the assumptions of twin modeling: namely (i) that phenotypic correlations and variances are the same whether an individual is a member of an MZ or a DZ pair, and whether the individual is arbitrarily assigned to be twin 1 or twin 2 in the model; (ii) in addition, within MZ and DZ pairs, cross-twin cross-trait correlations are constrained to be independent of order (e.g. the correlation between ADHDtwin1 and RTVtwin2 = the correlation between ADHDtwin2 and RTVtwin1).

Biometrical genetic modeling is based on three assumptions: (1) MZ twins share 100% of their segregating alleles and DZ twins share 50% of additive genetic (A) influences, but only 25% of non-additive genetic influences (D); (2) for twin pairs reared together, both members of MZ and DZ twin pairs are 100% concordant for their shared environmental (C) influences; and (3) child- or individual-specific environmental factors (E; which subsume any measurement error) do not contribute to the similarity between twin pairs. From this we can derive the following within-pair twin correlation expectations: (1) additive genetic influences (A) will double the MZ twin pair correlation in relation to the DZ twin pair correlation; (2) non-additive genetic influences (D) will more than double the MZ twin pair correlation in relation to the DZ twin pair correlation; (3) shared environmental effects (C) will increase within-pair MZ and DZ correlations to the same extent, reflected by DZ correlations that are more than half the MZ correlations; and (4) non-shared environment (E) will decrease both MZ and DZ correlations, most commonly identified in MZ correlations that are less than 1.

Using the same logic outlined above, multivariate genetic models use the MZ:DZ ratio of cross-twin cross-trait correlations to additionally estimate the extent to which the correlations between traits are caused by A, C and E influences. A Cholesky (triangular) decomposition is fit to the data, but to avoid giving precedence to the first measured variable in the model (which is arbitrary), a correlated factors solution of the Cholesky model is interpreted (Loehlin, 1996). This gives an estimate of the relative strengths of A, C and E for each trait and an estimation of the extent to which traits share their underlying etiological variance components through genetic (*r*_A_), shared environmental (*r*_C_) and child-specific environmental (*r*_E_) correlations.

For ADHD, an ADE model was the best fit, but for cognitive variables there was some, though very little, non-significant influence of C. Given power issues with distinguishing between A and D, and the non-significant C components, AE model results are presented for all multivariate analyses, which did not show a decrease in fit compared to the full model. The multivariate models included a scaling factor to account for male and female variance differences. No other quantitative and qualitative sex differences in genetic parameters were indicated.

#### Modeling ADHD and control sibling-pair data

Both phenotypic and familial structural equation models were run in Mx. Constrained phenotypic correlation models were run to estimate sibling correlations that were corrected for sample ascertainment and the familial clustering within the dataset. Constraints (inherent to the genetic modeling) are applied to give: (i) one set of correlations between traits within siblings, regardless of affection status; (ii) sibling correlations for each trait apart from ADHD, which is fixed to 0.40; and (iii) one set of cross-sibling cross-trait correlations.

Familial models were run in a similar manner as the genetic models, with two exceptions: (1) siblings, like DZ twins, share 50% of their segregating alleles and 100% of the shared environmental influences. Without a comparison group (such as MZ twins) it is impossible to exactly estimate A. Therefore, A and C are modeled together as one ‘familial’ factor (F). This parameter reflects all the contribution of C to the phenotypic similarity between siblings and 50–100% of A, such that if the variance in a trait was all due to C, F would be an exact estimate, but if C only partially underlies a trait, F becomes more conservative as the amount of C decreases. Thus, F estimates on this sample may be comparable to A estimates from the twin sample, but are likely to be conservative. (2) The selected nature of the sample was accounted for by including ADHD (the selection variable) in all models and fixing its parameters to known values. This necessitated ordinal analysis as ADHD status was coded categorically: affected/subthreshold/unaffected. Ordinal analysis assumes that the ordered categories reflect measurement of an underlying normal distribution of the trait. This liability distribution has one or more thresholds that distinguish between the categories. The familiality of ADHD was fixed to 40%, representing 80% genetic variance, as expected by population norms (Nikolas & Burt, 2010), and the thresholds on the ADHD liability were fixed to *z* values of 1.64 to give a population prevalence of 5%, and to 1.87 to reflect the ‘subthreshold’ position. Similar threshold fixes are required in the constrained phenotypic correlation models described above (with the sibling correlation for ADHD = 0.40). This method for correcting for sample ascertainment has been previously validated (Rijsdijk *et al.* 2005).

## Results

Table 1 provides the definitions of RTV variables, Table 2 shows the means and standard deviations for the cognitive data and Table 3 the genetic parameters. Because of multicollinearity, rather than one multivariate model across all traits, several smaller trivariate (correlations and genetic) models were run on ADHD or ADHD symptom scores, RTV (s.d. of RTs) in each condition and RTV difference scores between conditions. This meant that several parameters were estimated in more than one model. These differed only slightly (always *p* < 0.05), so we present the most conservative estimates in these cases.

**Table 2.** Means (and standard deviations) for RTV for MZ and DZ twins, ADHD probands, siblings of probands and controls

	Population-based twin sample		ADHD and control sibling-pair sample		
	MZ twins	DZ twins	ADHD probands	Siblings of probands	Controls
Go/no-go task					
RTV_slow_	218.31 (139.40)	224.37 (153.59)	312.79 (221.37)	225.48 (169.37)	143.54 (103.73)
RTV_fast_	166.17 (59.45)	157.78 (56.40)	143.26 (67.81)	187.49 (72.87)	115.77 (57.77)
RTV_incentive_	146.44 (75.90)	143.73 (68.20)	144.01 (91.98)	188.85 (129.27)	144.02 (55.01)
RTV-dif_slow-fast_	52.02 (134.84)	67.25 (144.75)	78.21 (141.26)	121.45 (199.58)	28.56 (98.36)
RTV-dif_slow-incentive_	71.55 (134.75)	81.13 (151.76)	55.95 (144.09)	87.74 (184.19)	25.63 (97.94)
Fast task					
RTV_baseline_	413.47 (281.78)	412.03 (302.23)	455.39 (343.55)	357.82 (323.58)	202.58 (178.50)
RTV_FI_	213.55 (165.61)	202.46 (129.54)	204.70 (180.60)	229.78 (156.82)	122.99 (102.01)
RTV-dif_baseline-FI_	199.87 (258.30)	209.10 (274.25)	150.38 (243.40)	234.15 (287.44)	79.52 (139.87)

RTV, Reaction time variability; MZ, monozygotic; DZ, dizygotic; ADHD, attention deficit hyperactivity disorder; dif, difference score; FI, fast incentive.

**Table 3.** Maximum-likelihood across-trait, across-twin and across-sibling correlations for RTV between each condition and the difference scores (constrained correlation models), and corresponding r_*A*_, r_*F*_ and r_*E*_ estimates (standardized solution of the genetic models)

	Population-based twin sample					ADHD and control sibling-pair sample			
	Cross-trait correlations			Genetic model		Cross-trait correlations		Genetic model	
	Within-individuals	Cross-twins MZ (*r*_MZ_)	Cross-twins DZ (*r*_DZ_)	*r*_A_	*r*_E_	Within-individuals	Cross-siblings	*r*_F_	*r*_E_
Go/no-go task									
RTV_slow_ and	**0.78**	**0.21**	**0.09**	**0.85**	**0.79**	**0.83**	**0.17**	**0.82**	0.83
RTV-dif_slow-incentive_	**(0.76 to 0.80**)	**(0.12 to 0.31**)	**(0.01 to 0.18**)	**(0.73 to 0.98**)	**(0.75 to 0.82**)	**(0.79 to 0.85**)	**(0.06 to 0.27**)	**(0.53 to 0.95**)	(0.78 to 0.87)
RTV_slow_ and	**0.82**	**0.25**	**0.11**	**0.81**	**0.84**	**0.90**	**0.15**	**0.78**	0.92^a^
RTV-dif_slow-fast_	**(0.81 to 0.84**)	**(0.15 to 0.34**)	**(0.01 to 0.20**)	**(0.72 to 0.88**)	**(0.81 to 0.87**)	**(0.89 to 0.91**)	**(0.04 to 0.24**)	**(0.50 to 0.79**)	(0.92 to 0.92)
RTV_incentive_ and	**−0.19**	0.07	0.07	**0.51**	**−0.37**	**−0.10**	**−0.01**	−0.07	−0.11
RTV-dif_slow-incentive_	**(−0.24 to −0.14**)	(0.00 to 0.16)	(0.00 to 0.15)	**(0.13 to 1.00**)	**(−0.46 to −0.28**)	**(−0.19 to −0.01**)	**(−0.11 to 0.08**)	(−0.58 to 0.52)	(−0.24 to 0.03)
RTV_incentive_ and	**−0.17**	0.04	−0.01	0.16	**−0.31**	−0.02	0.04	0.20	−0.09
RTV-dif_slow-fast_	**(−0.22 to −0.11**)	(−0.02 to 0.12)	(−0.08 to 0.06)	(−0.09 to 0.46)	**(−0.41 to −0.21**)	(−0.09 to 0.06)	(−0.04 to 0.12)	(−0.16 to 0.61)	(−0.20 to 0.03)
RTV-dif_slow-incentive_ and	**0.75**	**0.18**	0.08	**0.81**	**0.74**	**0.79**	**0.13**	**0.83**	**0.78**
RTV-dif_slow-fast_	**(0.72 to 0.77**)	**(0.06 to 0.28**)	(−0.01 to 0.16)	**(0.62 to 0.93**)	**(0.70 to 0.78**)	**(0.75 to 0.82**)	**(0.03 to 0.22**)	**(0.38 to 1.00**)	**(0.71 to 0.83**)
Fast task									
RTV_baseline_ and	**0.72**	**0.37**	**0.10**	**0.98**	**0.61**	**0.85**	**0.27**	**0.93**	**0.83**
RTV-dif_baseline-FI_	**(0.69 to 0.75**)	**(0.28 to 0.45**)	**(0.01 to 0.20**)	**(0.90 to 1.00**)	**(0.55 to 0.67**)	**(0.85 to 0.87**)	**(0.20 to 0.30**)	**(0.87 to 1.00**)	**(0.79 to 0.83**)
RTV_incentive_ and	**−0.07**	**0.20**	**0.12**	**0.76**	**−0.37**	**0.25**	**0.16**	**0.64**	0.12
RTV-dif_baseline-FI_	**(−0.12 to −0.01**)	**(0.12 to 0.31**)	**(0.04 to 0.20**)	**(0.45 to 0.98**)	**(−0.45 to −0.27**)	**(0.17 to 0.33**)	**(0.07 to 0.25**)	**(0.29 to 1.00**)	(0.00 to 0.25)

RTV, Reaction time variability; MZ, monozygotic; DZ, dizygotic; ADHD, attention deficit hyperactivity disorder; dif, difference score; FI, fast incentive.

a Confidence intervals for this estimate could not be calculated.

Significant (*p* < 0.05) estimates in bold; non-significant in normal typeface.

95% confidence intervals given in parentheses.

### Go/no-go task results

In this study we focus on the across-trait, across-twin and across-sibling correlations for RTV between each condition and the RTV difference scores, and corresponding across *r*_A_, r_F_ and *r*_E_ estimates (Table 3).

The majority of within-individual (phenotypic) correlations were not significantly different between the general population and selected clinical samples (as indicated by overlapping 95% confidence intervals), suggesting that comparisons across the two populations are appropriate. RTV_slow_ showed high phenotypic (0.78 and 0.83) and genetic/familial (0.85 and 0.82) correlations with RTV-dif_slow-incentive_ in both samples (Table 3). The same pattern of results was seen for the relationship with RTV-dif_slow-fast_. By contrast, the phenotypic correlations between the RTV_incentive_ condition with both RTV-dif_slow-fast_ and RTV-dif_slow-incentive_ were low (−0.02 to −0.19), with low-to-moderate genetic/familial correlations (most of which were non-significant). A direct comparison across go/no-go task RTV-dif_slow-fast_ and RTV-dif_slow-incentive_ indicated high phenotypic (0.75 and 0.79) and genetic/familial (0.81 and 0.83) correlations (Table 3).

### Fast task results

The fast task showed a similar pattern of results. RTV_baseline_ showed high phenotypic (0.72 and 0.85) and genetic/familial (0.98 and 0.93) correlations with RTV-dif_baseline-FI_ (Table 3). By contrast, the phenotypic correlations between RTV_incentive_ and RTV-dif_baseline-FI_ were low (−0.07 and 0.25); the genetic/familial correlations were moderately high (0.76 and 0.64) but need to be interpreted in light of the low phenotypic correlations, as there was no or limited phenotypic association to account for.

## Discussion

The RTV across-condition difference scores can be seen as an index of an individual's potential for reducing RTV under certain task conditions (fast, rewarded). Our results show that these difference scores measure largely the same underlying etiological process as RTV under baseline (slow, unrewarded) conditions on the go/no-go and fast tasks. The findings were replicated across a clinical combined subtype ADHD and control sibling-pair sample and population-based twin sample, across clinical diagnostic and quantitative trait approaches, and across tasks and different task manipulations. By contrast, RTV under rewarded and/or fast conditions measures a partly distinct process, indexing ‘the best performance one is capable of’.

On the go/no-go task, we observed high phenotypic correlations (0.78 and 0.83) between RTV under the slow condition and a difference score in RTV from the slow to the incentive condition, in both samples. RTV under the slow condition correlated, at the phenotypic level, equally highly (0.82 and 0.90) with RTV difference scores from the slow to the fast condition. Furthermore, the genetic model fitting analyses indicated shared etiology between RTV under the slow condition with either RTV difference score, with 78–85% of the familial/genetic influences shared between the two variables. By contrast, RTV under the fast condition showed no or low negative phenotypic correlations with the RTV slow-to-fast condition difference score, and low familial/genetic and individual-specific correlations. We obtained nearly identical results with RTV under the incentive condition, with low, negative phenotypic correlations with RTV difference scores from the slow to the incentive condition, and low to moderate genetic/familial correlations.

These findings indicate that there is little in the RTV difference scores that is not already captured by RTV baseline scores. Given possible psychometric disadvantages of difference scores (Peter *et al.* 1993), our findings suggest that, for most analyses, including genetic analyses, RTV baseline scores should be selected in preference to RTV difference scores. This is also advantageous for large collaborative genetic studies, where data across similar but not identical cognitive tasks are combined across projects, as in most studies only baseline RTV data are available.

Findings on the fast task further confirmed this pattern. The phenotypic correlations between RTV in a baseline condition and RTV difference from baseline to the fast-incentive condition were high (0.72 and 0.85) in both samples. Nearly all (93–98%) genetic/familial influences between these variables were shared. Low phenotypic correlations were again observed between RTV in the fast-incentive condition and the difference in RTV from the baseline to the fast-incentive condition (−0.07 and 0.25). The genetic/familial correlations were higher, but need to be interpreted cautiously, given the limited (and in one case, non-significant) extent of an observable association that they account for.

The theoretical implication of the finding of RTV baseline performance measuring the same process as the RTV difference scores is the support for theories that emphasize the malleability of the observed high RTV, such as the models incorporating an arousal regulation process in ADHD (van der Meere, 2002; Sergeant, 2005; Johnson *et al.* 2007; Halperin *et al.* 2008; O'Connell *et al.* 2009). Further theoretical insight is obtained from the finding of a high degree of overlap in the phenotypic, genetic/familial and individual-specific environmental association (with correlations of 0.74–0.83) between the two different RTV difference scores (slow-fast and slow-incentive) from the go/no-go task. Although, in ADHD, the association with diagnosis is slightly greater with the RTV slow-incentive difference score than with the slow-fast difference score (Kuntsi *et al.* 2009; Uebel *et al.* 2010), our findings here indicate that both difference scores measure, to a large part, the same underlying process.

The psychometric properties of difference scores have been the subject of much investigation and debate (Cronbach & Furby, 1970; Johns, 1981; Peter *et al.* 1993). One shortcoming of difference scores is that the reliability of the difference score is dependent on the reliability of both of the component scores; as they each decrease, so does the reliability of the difference score (Johns, 1981). Overall, the statistical shortcomings of difference scores further support our conclusion that, given the evidence for high etiological sharing between baseline and difference scores, RTV baseline scores should be selected in preference to RTV difference scores for most analyses. We note that the computational demands of ordinal data analysis, in conjunction with the correlated nature of some of the variables, precluded the full optimization of all confidence intervals. Point estimates should not have been affected, but the confidence intervals should be treated with caution.

Although our finding that high RTV also indicates greater potential for a decrease in RTV might seem unsurprising, it is not self-evident. If high RTV on tasks, such as the go/no-go and fast tasks, reflected a stable impairment, we would not observe an improvement in performance with rewards or with a faster event rate, and hence analyses of the kind presented here could not be performed. The implication is that, if disorders exist where no improvement in RTV is observed across conditions (a hypothetical scenario not observed or investigated in the current analyses), the current conclusions would not apply. The investigation of the sensitivity of RTV to reward and event rate manipulations in disorders such as schizophrenia or dementia is an important direction for future research. With regard to the samples studied in the present analyses, we note that the replication of the findings from the clinical sample to the population-based twin sample emphasizes how the phenomenon is not restricted to clinically defined samples. The extent of replication across the two samples is indicative of the robustness of the findings, given the differences in sample selection, age and gender composition.
